# Associations Between Low-Density Lipoprotein Cholesterol Levels and Cardiovascular Outcomes in Patients Undergoing Dialysis: A Nationwide Cohort Study

**DOI:** 10.3390/jcm14144845

**Published:** 2025-07-08

**Authors:** Byung Sik Kim, Jiyeong Kim, Nayeon Choi, Hyun-Jin Kim, Jeong-Hun Shin

**Affiliations:** 1 Division of Cardiology, Department of Internal Medicine, Hanyang University Guri Hospital, Hanyang University College of Medicine, Guri 11923, Republic of Korea; fish3777@hanmail.net (B.S.K.); titi8th@gmail.com (H.-J.K.); 2 Biostatistics Lab, Medical Research Collaborating Center, Industry-University Cooperation Foundation, Hanyang University, Seoul 04763, Republic of Korea; kimzi@hanyang.ac.kr (J.K.); nayeon@hanyang.ac.kr (N.C.); 3 Department of Pre-Medicine, College of Medicine, Hanyang University, Seoul 04763, Republic of Korea

**Keywords:** low-density lipoprotein cholesterol, dialysis, statin, outcome, cardiovascular disease

## Abstract

**Background/Objectives:** Low-density lipoprotein cholesterol (LDL-C) is a causal factor in the development of atherosclerosis and a predictor of cardiovascular disease. However, the association between LDL-C levels and cardiovascular outcomes in patients undergoing dialysis remains controversial, with current guidelines advising against initiating statin therapy in this population. This study investigated the relationship between LDL-C levels and cardiovascular outcomes in Korean adults undergoing dialysis, using nationwide data. **Methods**: A total of 21,692 patients with end-stage kidney disease undergoing dialysis between 2009 and 2017 were identified from the Korean National Health Insurance Service database. Statin non-users (primary cohort) and users (secondary cohort) comprised 15,414 and 6278 patients, respectively. LDL-C levels were categorized, and cardiovascular outcomes including composites of cardiovascular death, myocardial infarction, and ischemic stroke were analyzed. **Results**: Among statin non-users, LDL-C levels > 100 mg/dL were significantly associated with an increased risk of the composite outcome, in a dose-dependent manner, compared with LDL-C levels < 70 mg/dL. Specifically, participants with LDL-C levels ≥ 160 mg/dL demonstrated a 43% increased risk of the composite outcome and a 2.25-fold higher risk of myocardial infarction compared to those with LDL-C levels < 70 mg/dL. Among statin users, LDL-C levels > 130 mg/dL were associated with an increased risk of the composite outcome. **Conclusions**: This study highlights the significant association between elevated LDL-C levels and adverse cardiovascular outcomes in patients undergoing dialysis. These findings underscore the importance of close monitoring and proactive management of LDL-C levels in this high-risk population. Future research should focus on developing tailored lipid-lowering strategies to improve cardiovascular outcomes in these patients.

## 1. Introduction

Low-density lipoprotein cholesterol (LDL-C) is a well-established causal factor for atherosclerotic cardiovascular disease (ASCVD), which remains the leading global cause of mortality [1,2]. Lipid-lowering therapy is essential for the secondary prevention of ASCVD, regardless of baseline LDL-C levels, with increasing emphasis placed on achieving target LDL-C levels in clinical practice [3,4]. For primary prevention, tailoring lipid-lowering therapy according to an individual’s cardiovascular risk is also important [5,6]. Statins, which are supported by robust evidence for primary and secondary prevention, play a central role in lipid management [6,7].

Patients with chronic kidney disease (CKD) are at a particularly high risk of ASCVD, and this risk escalates significantly as the estimated glomerular filtration rate (eGFR) declines [8]. Statin-based therapy reduces the risk of ASCVD in patients with CKD [9]. Therefore, clinical guidelines recommend statin-based lipid-lowering therapies to reduce ASCVD risk [3,4,10]. However, in patients requiring maintenance dialysis, statin therapy has failed to demonstrate benefits in preventing ASCVD; thus, the current guidelines advise against initiating statin therapy in this population [11,12,13]. Nevertheless, some studies have reported potential benefits of statin therapy in patients undergoing dialysis [14]. Moreover, given the established direct causal relationship between LDL-C and atherosclerosis [15], the association between LDL-C levels and the risk of ASCVD in this high-risk population is not easily dismissed [16]. This raises critical questions regarding the role of LDL-C in the risk of ASCVD in patients on dialysis.

This study investigated the relationship between LDL-C levels and cardiovascular outcomes in Korean adults undergoing dialysis, using big data from the Korean National Health Insurance Service (NHIS) to provide insights into ASCVD risk in this high-risk population.

## 2. Materials and Methods

### 2.1. Data Sources and Study Population

This study utilized data from the NHIS database in South Korea, which provides comprehensive health-related information for the Korean population. The NHIS serves as a single public insurance provider, covering approximately 97% of the population, with the remaining 3% supported by the Medical Aid Program. The NHIS database includes detailed records of medical information, including diagnoses coded using the International Classification of Diseases, Tenth Revision (ICD-10), prescriptions, and outcomes such as mortality. Additionally, it incorporates data from biannual health screening examinations, including self-reported questionnaires, physical measurements, and laboratory test results. The NHIS database has previously been described in detail [17].

**Figure 1:**
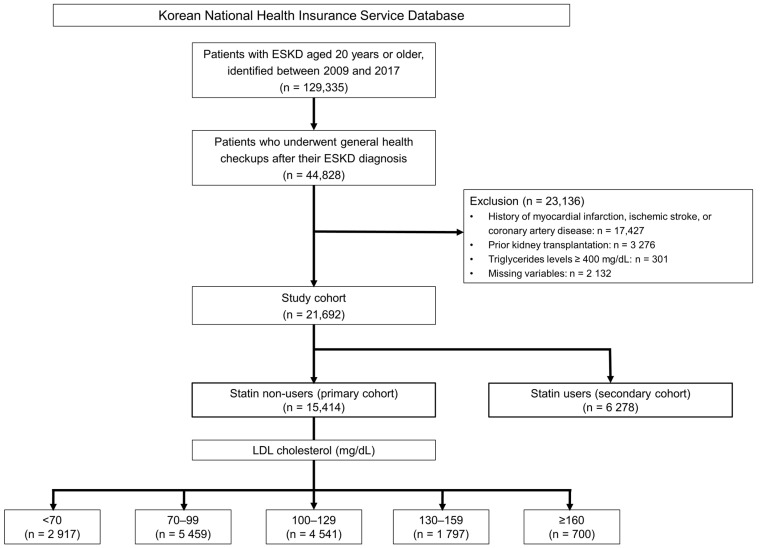
Flowchart illustrating the study population selection. ESKD, end-stage kidney disease; LDL, low-density lipoprotein.

A flowchart summarizing the selection process and subgroup classification is shown in Figure 1. The initial cohort included 129,335 patients with end-stage kidney disease (ESKD) aged ≥ 20 years, identified between 2009 and 2017. To define the study population, we identified patients prescribed mandatory procedure codes for dialysis (hemodialysis: O7020, O7021, and O9991; peritoneal dialysis: O707) for >90 consecutive days [18]. Among them, 44,828 patients who underwent general health checkups after ESKD diagnosis were selected for further analysis. The exclusion criteria were as follows: a history of ASCVD, including myocardial infarction (MI), ischemic stroke, or coronary artery disease (CAD) (*n* = 17,427); prior kidney transplantation (*n* = 3276); triglyceride (TG) levels ≥ 400 mg/dL (*n* = 301); and missing data (*n* = 2132). After applying these exclusion criteria, the final study cohort comprised 21,692 individuals. This cohort was further divided into two groups based on statin use: 15,414 statin non-users (primary cohort) and 6278 statin users (secondary cohort). Within the primary cohort, the participants were stratified into five subgroups based on their LDL-C levels: <70 mg/dL (*n* = 2917), 70–99 mg/dL (*n* = 5459), 100–129 mg/dL (*n* = 4541), 130–159 mg/dL (*n* = 1797), and ≥160 mg/dL (*n* = 700).

### 2.2. Data Collection

The baseline data in this study were defined as those recorded during the index general health checkup. The demographic variables included the participants’ age and sex. Systolic blood pressure (SBP) and diastolic blood pressure (DBP) were also measured. The assessed lifestyle factors included smoking status (never, past, or current), alcohol consumption frequency (0, 1–2, or ≥3 times per week), and exercise frequency (0, 1–2, 3–4, or ≥5 times per week). Household income was stratified into quartiles to account for socioeconomic status. Anthropometric and laboratory data included body mass index (BMI) (<18.5, 18.5–22.9, 23–24.9, or ≥25 kg/m^2^) and fasting glucose levels (<100, 100–125.9, or ≥126 mg/dL). LDL-C levels were calculated using the Friedewald formula based on the lipid profiles [19]. Medical history included the presence of hypertension (HTN) and diabetes mellitus (DM), as well as comorbidities assessed using the Charlson Comorbidity Index (≤3, 4, 5, 6, or ≥7 comorbidities) [20]. Data on the use of statins and other lipid-lowering drugs, as well as antihypertensive, antidiabetic, and antiplatelet agents, were also collected. Table S1 provides detailed definitions of the study population, comorbidities, medication history, and outcomes, including the diagnostic codes, procedure codes, and drug codes used for classification.

### 2.3. Outcomes

The primary outcome of this study was a composite of cardiovascular death, myocardial infarction (MI), and ischemic stroke evaluated during a follow-up period extending to 31 December 2022 (median duration, 8.1 years). The secondary outcomes included individual components of the primary composite outcome that were analyzed separately. Mortality data, including the cause and date of death, were obtained from the National Statistical Office of Korea. Cardiovascular death was defined as death due to cardiovascular disease (*ICD-10* codes I00–I99) as recorded on death certificates. MI was defined as hospitalized patients who underwent coronary revascularization and were discharged with *ICD-10* codes I21 or I22. Ischemic stroke was defined as hospitalized patients who underwent brain imaging and had discharge diagnoses with *ICD-10* codes I63 or I64.

### 2.4. Statistical Analysis

Continuous variables are expressed as means ± standard deviations and were compared using one-way analysis of variance, whereas categorical variables are presented as frequencies and percentages and were analyzed using the chi-squared test. The incidence rates were calculated as the number of outcomes per 1000 person-years during the follow-up period. To evaluate the association between LDL-C levels and clinical outcomes, Kaplan–Meier curves were generated, and the event rates across LDL-C categories were compared using log-rank tests. Hazard ratios (HRs) and 95% confidence intervals (CIs) were estimated using Cox proportional hazards regression models, with LDL-C < 70 mg/dL serving as the reference category. Three models were constructed to adjust for potential confounders: Model 1 was adjusted for age and sex; Model 2 was further adjusted for BMI, smoking status, alcohol consumption, exercise, and household income; and Model 3 was further adjusted for Charlson Comorbidity Index, HTN, DM, and antiplatelet agent use. Restricted cubic spline curve analysis was performed to visualize the continuous relationship between LDL-C levels and clinical outcomes after adjusting for the covariates included in Model 3. All statistical analyses were performed using SAS Enterprise version 7.1 (SAS Inc., Cary, NC, USA) and RStudio version 4.0.3 (The R Foundation, Vienna, Austria). All *p*-values were two-sided, with values < 0.05 considered statistically significant.

## 3. Results

### 3.1. Baseline Characteristics

Table 1 summarizes the baseline characteristics of the primary cohort (statin non-users undergoing dialysis), stratified by LDL-C category. Among the 15,414 participants, the largest proportion was in the 70–99 mg/dL group (35.4%), whereas the smallest group had LDL-C levels ≥ 160 mg/dL (4.5%). Significant differences in age, sex, smoking status, alcohol consumption, BMI, fasting glucose levels, and comorbidities were observed across the LDL-C categories (*p* < 0.001). Participants with higher LDL-C levels tended to be older, with a greater proportion of females, a lower smoking prevalence, and a higher BMI. The prevalence of DM and fasting blood glucose levels also increased with increasing LDL-C levels. However, blood pressure and exercise frequency showed minimal variation across the groups.

### 3.2. Clinical Outcomes According to LDL-C Categories in the Primary Cohort (Statin Non-Users)

**Figure 2:**
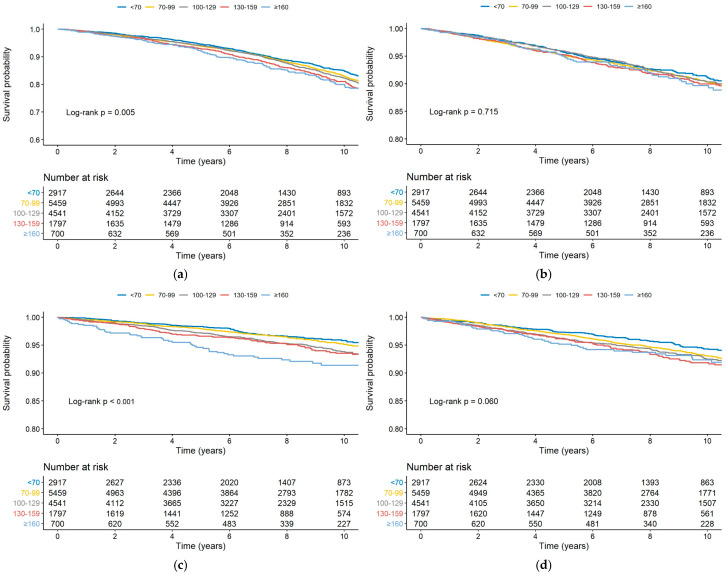
Kaplan–Meier survival curves for the primary cohort, stratified by LDL-C categories: (**a**) Composite outcome, including cardiovascular death, myocardial infarction, and ischemic stroke. (**b**) Cardiovascular death. (**c**) Myocardial infarction. (**d**) Ischemic stroke. LDL-C, low-density lipoprotein cholesterol.

Figure 2 illustrates the Kaplan–Meier survival curves for the primary cohort, stratified by LDL-C categories, and highlights the composite outcomes (Figure 2a), cardiovascular death (Figure 2b), MI (Figure 2c), and ischemic stroke (Figure 2d). The log-rank test showed significant differences in survival probabilities for the composite outcome (*p* = 0.005) and MI (*p* < 0.001), with higher LDL-C levels linked to progressively worse outcomes.

Table 2 summarizes the HRs derived from the Cox proportional hazards regression analysis across LDL-C categories for clinical outcomes. Sequential adjustments were made in the three models to account for potential confounders. In the fully adjusted models, the LDL-C categories of 100–129, 130–159, and ≥160 mg/dL were significantly associated with higher HRs for the composite outcome. Notably, participants with LDL-C levels ≥ 160 mg/dL demonstrated a 43% increased risk compared to those with LDL-C levels < 70 mg/dL (HR: 1.43, 95% CI: 1.16–1.75, *p* < 0.001). While cardiovascular death showed a trend of increasing HRs with increasing LDL-C levels, these differences were not statistically significant. Meanwhile, MI exhibited a clear association, with higher LDL-C levels linked to progressively greater risk across all groups with LDL-C ≥ 70 mg/dL than in those with LDL-C < 70 mg/dL. In particular, participants with LDL-C levels ≥ 160 mg/dL demonstrated a 125% increased risk compared to those with LDL-C levels < 70 mg/dL (HR: 2.25, 95% CI: 1.58–3.20, *p* < 0.001). Ischemic stroke also showed a statistically significant trend of an increased risk with higher LDL-C levels.

**Figure 3:**
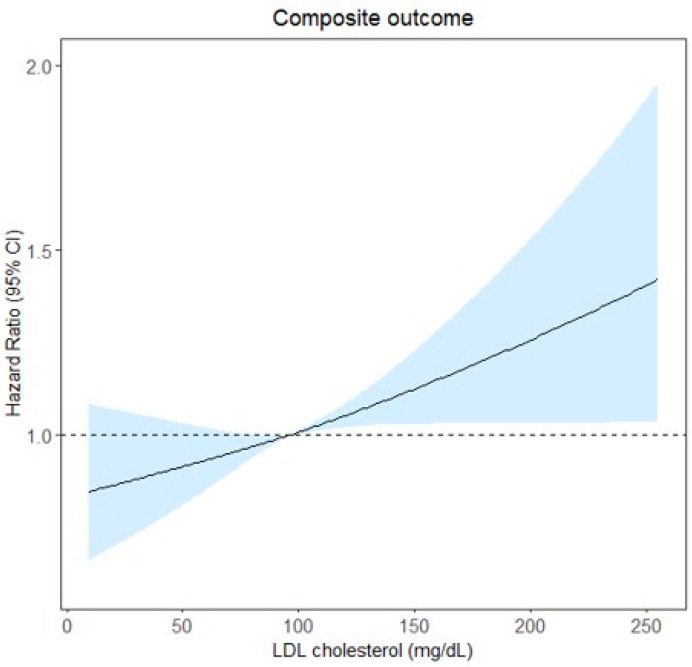
Restricted cubic spline curves illustrating the continuous relationships between LDL-C levels and composite cardiovascular outcome, including cardiovascular death, myocardial infarction, and ischemic stroke. LDL-C, low-density lipoprotein cholesterol.

Restricted cubic spline curves illustrating the continuous relationship between LDL-C levels and the composite outcome are shown in Figure 3, while the associations with individual outcomes are presented in Figure S1: cardiovascular death (Figure S1A), MI (Figure S1B), and ischemic stroke (Figure S1C). As LDL-C levels increased, the composite outcome showed a trend of increasing risk, with the strongest association observed for MI. In contrast, the association with cardiovascular death was the least pronounced.

### 3.3. Clinical Outcomes According to LDL-C Categories in the Secondary Cohort (Statin Users)

Table 3 summarizes the HRs derived from the Cox regression analysis across LDL-C categories for clinical outcomes in the secondary cohort. Statin users demonstrated relatively lower LDL-C levels, with 74.2% of the participants having LDL-C levels < 100 mg/dL. The associations with clinical outcomes were less pronounced than those in the primary cohort but generally exhibited similar trends. In the fully adjusted models, LDL-C levels of 130–159 mg/dL and ≥ 160 mg/dL were significantly associated with higher HRs for the composite outcome, with participants having LDL-C levels ≥ 160 mg/dL showing a 56% increased risk compared to those with LDL-C < 70 mg/dL (HR: 1.56, 95% CI: 1.12–2.18, *p* = 0.009). Cardiovascular death did not differ significantly between the groups. While ischemic stroke demonstrated a trend of increasing HRs with higher LDL-C levels, these differences were not statistically significant. In contrast, MI exhibited a clear association, with higher LDL-C levels linked to progressively greater risk across all groups with LDL-C ≥ 70 mg/dL compared with those with LDL-C < 70 mg/dL. Participants with LDL-C levels ≥ 160 mg/dL demonstrated a 125% increased risk compared to those with LDL-C levels < 70 mg/dL (HR: 2.25, 95% CI: 1.58–3.20, *p* < 0.001).

As a sensitivity analysis, we reclassified the LDL-C categories to include a <55 mg/dL group. The risks of the primary composite outcome and individual outcomes were generally comparable to those in the main analysis (Tables S2 and S3).

## 4. Discussion

This study investigated the association between LDL-C levels and cardiovascular outcomes in patients undergoing dialysis, using nationwide data from the Korean NHIS. The key findings were as follows: (1) LDL-C levels > 100 mg/dL were associated with a significantly increased risk of composite cardiovascular outcomes, in a dose-dependent manner, among statin non-users; (2) notably, statin non-users with LDL-C levels ≥ 160 mg/dL had a 43% increased risk of the composite cardiovascular outcome and a 2.25-fold higher risk of MI compared to those with LDL-C levels < 70 mg/dL; and (3) in the secondary cohort of statin users, LDL-C levels > 130 mg/dL were also associated with an increased risk of the composite outcome. This is the first large-scale cohort study to demonstrate an association between high LDL-C levels and adverse cardiovascular outcomes in statin non-users undergoing dialysis. These findings underscore the importance of recognizing the risks associated with high LDL-C levels in this high-risk population and highlight the need for tailored therapeutic strategies to mitigate the risk of ASCVD.

LDL-C demonstrates a well-established linear association with cardiovascular outcomes in the general population and patients with CKD; however, this relationship diminishes as the eGFR declines, despite the higher absolute risk of ASCVD events [21]. Among patients undergoing dialysis, the evidence fails to support a positive association between elevated LDL-C levels and cardiovascular outcomes, with some studies indicating a potentially inverse relationship [22]. Nevertheless, the efficacy of statins in broader populations has prompted trials to investigate their potential benefits in this high-risk group [23]. Two large, randomized controlled trials—the Die Deutsche Diabetes Dialysis Studies (4D), which included 1255 patients with diabetes on hemodialysis, and A Study to Evaluate the Use of Rosuvastatin in Subjects on Regular Hemodialysis: An Assessment of Survival and Cardiovascular Events (AURORA), which included 2776 patients on hemodialysis—found no cardiovascular benefit of statin therapy in patients on hemodialysis, despite significant LDL-C reductions [11,12]. In contrast, the Study of Heart and Renal Protection (SHARP), which included 9270 patients with CKD, demonstrated a clear benefit of LDL-C reduction in atherosclerotic cardiovascular events. However, this benefit was not observed in the subgroup of patients undergoing dialysis at the time of enrolment [13]. Based on these studies, the current guidelines strongly advocate statin-based lipid-lowering treatments in patients with CKD to rigorously mitigate ASCVD risk. However, in dialysis-dependent patients with CKD, statins or statin combinations should not be initiated, although continuation is advised for those already receiving such treatments at the time of dialysis initiation [3,4,10].

While previous randomized studies failed to demonstrate the benefit of LDL-C-lowering treatment in patients on dialysis, this finding cannot be definitively concluded and should be interpreted given the following considerations: First, the inclusion criteria did not specifically target patients with elevated LDL-C levels, which could have attenuated the observed benefits of LDL-C-lowering treatments. Second, as the benefit of lowering LDL-C decreases with deteriorating kidney function, the relatively small sample sizes of patients on dialysis in these trials compared to other major statin trials [24] may have limited the statistical power to detect significant effects. Lastly, and most importantly, considering the well-established causal relationship between LDL-C and atherosclerosis, a lack of this association in patients undergoing dialysis is unlikely [15]. Pathophysiologically, LDL-C is the primary driver of atherogenesis, promoting cholesterol accumulation, foam cell formation, and a pro-inflammatory, prothrombotic environment that contributes to plaque necrosis [25]. In addition, strong and consistent evidence from genetic studies, prospective cohorts, and randomized trials confirms that LDL-C is not merely a risk marker but a causal factor in the development of ASCVD [15]. Therefore, large-scale population-based studies are needed to definitively evaluate the association between LDL-C levels and ASCVD risk in patients on dialysis. The findings of this study provide crucial insights in this context. The secondary cohort of statin users demonstrated the benefits of LDL-C reduction, particularly in mitigating the risk of MI. More importantly, in the primary cohort of statin non-users, for whom statin therapy is not typically recommended under the current guidelines, LDL-C levels were more strongly associated with adverse cardiovascular outcomes. Notably, participants with LDL-C levels ≥ 100 mg/dL exhibited significantly higher risks of MI, ischemic stroke, and composite outcomes compared to those with LDL-C levels < 70 mg/dL, emphasizing the need to revisit and refine therapeutic strategies for this high-risk population.

Although our study demonstrated significant associations between elevated LDL-C levels and the primary composite outcome, MI, and ischemic stroke, the association with cardiovascular death was not statistically significant. This finding likely reflects the multifactorial nature of mortality among dialysis patients, who often experience substantial burdens of non-atherosclerotic conditions such as systemic inflammation, protein energy wasting, vascular calcification, and arrhythmias, all contributing significantly to the risk of mortality beyond traditional lipid-mediated mechanisms [26]. Additionally, ESKD itself is associated with altered lipid metabolism and impaired response to lipid-lowering therapies, potentially diminishing the predictive value of LDL-C levels for cardiovascular mortality in this population [27]. These complex interactions may partially explain the attenuated association between LDL-C levels and cardiovascular death observed in our analysis.

The findings of this study highlight important considerations for lipid management in patients undergoing dialysis. In the context of the primary prevention of ASCVD, the current guidelines advise against initiating statin therapy in patients with ESKD, owing to insufficient evidence of benefits. However, this approach carries the potential risk of neglecting LDL-C management in one of the highest-risk populations, where the incidence of MI is strikingly 4–8 times greater than that in the general population [28]. Our results suggest that LDL-C levels remain an important marker of ASCVD in patients with ESKD. Specifically, participants with LDL-C levels ≥ 160 mg/dL demonstrated a 2.25-fold higher risk of MI compared to those with LDL-C levels < 70 mg/dL. These findings underscore the necessity of close monitoring of lipid profiles and implementation of lifestyle interventions to address LDL-C management, particularly in patients on dialysis with markedly elevated LDL-C levels. Moreover, the development of tailored therapeutic strategies for the management of LDL-C levels in high-risk populations is urgently needed. Future research should focus on defining the optimal LDL-C thresholds and treatment targets for patients with ESKD. Studies focusing exclusively on patients with high LDL-C levels are critical for reassessing the role of statins in this group. Additionally, the efficacy of non-statin therapies, alone or in combination with statins, such as ezetimibe, proprotein convertase subtilisin kexin type 9 inhibitors, bempedoic acid, or inclisiran, should be explored [29]. Large-scale randomized trials are essential to refine treatment guidelines and improve cardiovascular outcomes in this vulnerable population.

Despite its significant findings, this study has some limitations. First, the retrospective study design limited the ability to establish causal relationships between LDL-C levels and cardiovascular outcomes. Second, reliance on claims data introduces challenges such as variability in diagnostic coding and potential misclassification of comorbidities or outcomes. Although strict criteria were applied to improve the data accuracy, coding inaccuracies cannot be entirely ruled out. Third, although this study adjusted for numerous confounders, including demographic, lifestyle, and clinical variables, unmeasured factors such as dietary patterns, treatment adherence, and genetic predispositions may have influenced the observed associations. Additionally, our study was limited by the use of LDL-C levels calculated using the Friedewald formula rather than directly measured LDL-C, potentially affecting the accuracy of LDL-C estimation. Fourth, detailed information on statin dosage and combination therapies (e.g., statin plus ezetimibe) was not available, as medication data were primarily used for cohort classification rather than exposure analysis. Finally, this study used a nationwide database covering almost the entire Korean population to offer comprehensive insights. However, caution is required when applying these findings to other countries, ethnic groups, and healthcare systems, as differences in genetics, lifestyles, and medical practices may influence the results. Future prospective studies will be required to validate these findings.

## 5. Conclusions

This study demonstrates the significant association of elevated LDL-C levels with increased risks of adverse cardiovascular outcomes in patients undergoing dialysis. Future studies are warranted to explore the potential benefits of targeted lipid-lowering strategies in patients with elevated LDL-C levels undergoing dialysis.

## Figures and Tables

**Table 1:** Baseline characteristics of the primary cohort (statin non-users undergoing dialysis).

		Low-Density Lipoprotein Cholesterol Level (mg/dL)					
Total Patients (*n* = 15,414)		<70	70–99	100–129	130–159	≥160	*p*-Value
		(*n* = 2917)	(*n* = 5459)	(*n* = 4541)	(*n* = 1797)	(*n* = 700)	
Age, years		56.1 ± 11.9	57.2 ± 11.8	57.9 ± 11.7	58.3 ± 11.5	57.8 ± 10.9	<0.001
Sex, *n* (%)							<0.001
	Male	2193 (75.2)	3653 (66.9)	2668 (58.8)	892 (49.6)	300 (42.9)	
	Female	724 (24.8)	1806 (33.1)	1873 (41.2)	905 (50.4)	400 (57.1)	
Blood pressure, mmHg							
	SBP	136.3 ± 21.2	135.1 ± 19.5	135.3 ± 20.1	135.4 ± 21.4	135.2 ± 21.6	0.140
	DBP	79.7 ± 12.5	79.5 ± 12.1	79.9 ± 12.2	79.7 ± 12.3	80.6 ± 13.2	0.155
Fasting glucose, mg/dL		110.7 ± 53.2	108.2 ± 44.7	107.4 ± 41.8	114.2 ± 49.8	112.3 ± 44.2	<0.001
Smoking, *n* (%)							<0.001
	Never	1608 (55.1)	3291 (60.3)	2891 (63.7)	1229 (68.4)	515 (73.6)	
	Past	775 (26.6)	1401 (25.7)	1119 (24.6)	395 (22.0)	122 (17.4)	
	Current	534 (18.3)	767 (14.1)	531 (11.7)	173 (9.6)	63 (9.0)	
Alcohol consumption, times/week							<0.001
	0	2463 (84.4)	4818 (88.3)	4106 (90.4)	1633 (90.9)	652 (93.1)	
	1–2	345 (11.8)	513 (9.4)	347 (7.6)	128 (7.1)	40 (5.7)	
	≥3	109 (3.7)	128 (2.3)	88 (1.9)	36 (2.0)	8 (1.1)	
Exercise, times/week							0.366
	0	1849 (63.4)	3389 (62.1)	2861 (63.0)	1135 (63.2)	447 (63.9)	
	1–2	502 (17.2)	1009 (18.5)	770 (17.0)	329 (18.3)	111 (15.9)	
	3–4	331 (11.3)	602 (11.0)	558 (12.3)	199 (11.1)	79 (11.3)	
	≥5	235 (8.1)	459 (8.4)	352 (7.8)	134 (7.5)	63 (9.0)	
Body mass index, kg/m^2^							<0.001
	<18.5	244 (8.4)	362 (6.6)	258 (5.7)	85 (4.7)	24 (3.4)	
	18.5–22.9	1508 (51.7)	2838 (52.0)	2210 (48.7)	806 (44.9)	286 (40.9)	
	23–24.9	597 (20.5)	1182 (21.7)	1023 (22.5)	427 (23.8)	171 (24.4)	
	≥25	568 (19.5)	1077 (19.7)	1050 (23.1)	479 (26.7)	219 (31.3)	
Household income, quartiles							<0.001
	First	1154 (39.6)	1954 (35.8)	1527 (33.6)	607 (33.8)	231 (33.0)	
	Second	519 (17.8)	947 (17.3)	840 (18.5)	308 (17.1)	136 (19.4)	
	Third	621 (21.3)	1192 (21.8)	983 (21.6)	410 (22.8)	181 (25.9)	
	Fourth	623 (21.4)	1366 (25.0)	1191 (26.2)	472 (26.3)	152 (21.7)	
Charlson Comorbidity Index							<0.001
	≤3	808 (27.7)	1596 (29.2)	1458 (32.1)	535 (29.8)	211 (30.1)	
	4	435 (14.9)	893 (16.4)	742 (16.3)	319 (17.8)	117 (16.7)	
	5	447 (15.3)	893 (16.4)	746 (16.4)	285 (15.9)	112 (16.0)	
	6	410 (14.1)	729 (13.4)	609 (13.4)	234 (13.0)	104 (14.9)	
	≥7	817 (28.0)	1348 (24.7)	986 (21.7)	424 (23.6)	156 (22.3)	
Hypertension		2338 (80.2)	4326 (79.2)	3559 (78.4)	1453 (80.9)	559 (79.9)	0.170
Diabetes mellitus		1002 (34.4)	1812 (33.2)	1409 (31.0)	624 (34.7)	246 (35.1)	0.006
Antiplatelet agent use		1608 (55.1)	3045 (55.8)	2529 (55.7)	1025 (57.0)	354 (50.6)	0.061

DBP, diastolic blood pressure; SBP, systolic blood pressure.

**Table 2:** Clinical outcomes across LDL-C categories in the primary cohort (statin non-users).

Total Patients (*n* = 15,414)				Model 1		Model 2		Model 3	
LDL-C Categories	Participants	Events	Incidence Rate (per 1000 PY)	HR (95% CI)	*p*-Value	HR (95% CI)	*p*-Value	HR (95% CI)	*p*-Value
Composite outcome (cardiovascular death, myocardial infarction, and ischemic stroke)									
<70	2917	383	0.048	1 (Ref.)		1 (Ref.)		1 (Ref.)	
70–99	5459	823	0.054	1.07 (0.95–1.21)	0.273	1.09 (0.97–1.23)	0.166	1.12 (0.99–1.26)	0.076
100–129	4541	755	0.059	1.16 (1.02–1.31)	0.021	1.17 (1.04–1.33)	0.012	1.23 (1.08–1.39)	0.001
130–159	1797	316	0.063	1.26 (1.08–1.46)	0.003	1.29 (1.11–1.50)	0.001	1.32 (1.13–1.53)	<0.001
≥160	700	125	0.066	1.36 (1.11–1.66)	0.003	1.37 (1.12–1.68)	0.003	1.43 (1.16–1.75)	<0.001
Cardiovascular death									
<70	2917	202	0.025	1 (Ref.)		1 (Ref.)		1 (Ref.)	
70–99	5459	418	0.026	1.01 (0.86–1.20)	0.893	1.04 (0.88–1.23)	0.645	1.06 (0.90–1.25)	0.504
100–129	4541	353	0.026	0.99 (0.83–1.18)	0.898	1.02 (0.86–1.22)	0.795	1.06 (0.89–1.26)	0.548
130–159	1797	147	0.028	1.06 (0.86–1.32)	0.576	1.13 (0.91–1.40)	0.287	1.15 (0.92–1.42)	0.220
≥160	700	58	0.029	1.13 (0.85–1.52)	0.402	1.19 (0.89–1.60)	0.240	1.23 (0.91–1.65)	0.177
Myocardial infarction									
<70	2917	92	0.011	1 (Ref.)		1 (Ref.)		1 (Ref.)	
70–99	5459	202	0.013	1.12 (0.87–1.43)	0.371	1.13 (0.88–1.45)	0.337	1.16 (0.90–1.48)	0.248
100–129	4541	223	0.017	1.48 (1.16–1.90)	0.002	1.47 (1.15–1.87)	0.002	1.54 (1.21–1.97)	<0.001
130–159	1797	87	0.017	1.54 (1.14–2.07)	0.005	1.49 (1.10–2.00)	0.009	1.52 (1.13–2.04)	0.006
≥160	700	49	0.025	2.35 (1.66–3.33)	<0.001	2.21 (1.56–3.15)	<0.001	2.25 (1.58–3.20)	<0.001
Ischemic stroke									
<70	2917	131	0.016	1 (Ref.)		1 (Ref.)		1 (Ref.)	
70–99	5459	296	0.019	1.12 (0.91–1.38)	0.283	1.14 (0.92–1.40)	0.228	1.18 (0.96–1.44)	0.127
100–129	4541	275	0.021	1.21 (0.98–1.49)	0.080	1.21 (0.98–1.50)	0.070	1.29 (1.04–1.59)	0.019
130–159	1797	114	0.022	1.29 (1.00–1.66)	0.052	1.30 (1.01–1.68)	0.043	1.34 (1.04–1.73)	0.026
≥160	700	44	0.022	1.33 (0.95–1.88)	0.101	1.33 (0.94–1.88)	0.102	1.40 (0.99–1.98)	0.058

CI, confidence interval; HR, hazard ratio; LDL-C, low-density lipoprotein cholesterol; PY, person-years; Ref, reference. Model 1: adjusted for age and sex. Model 2: adjusted for age, sex, body mass index, smoking status, alcohol consumption, exercise, and household income. Model 3: adjusted for age, sex, body mass index, smoking status, alcohol consumption, exercise, household income, Charlson Comorbidity Index, hypertension, diabetes mellitus, and antiplatelet agent use.

**Table 3:** Clinical outcomes across LDL-C categories in the secondary cohort (statin users).

Total Patients (*n* = 6278)				Model 1		Model 2		Model 3	
LDL-C Categories	Participants	Events	Incidence Rate (per 1000 PY)	HR (95% CI)	*p*-Value	HR (95% CI)	*p*-Value	HR (95% CI)	*p*-Value
Composite outcome (cardiovascular death, myocardial infarction, and ischemic stroke)									
<70	2426	394	0.063	1 (Ref.)		1 (Ref.)		1 (Ref.)	
70–99	2234	375	0.064	1.03 (0.90–1.19)	0.659	1.04 (0.90–1.20)	0.619	1.10 (0.95–1.27)	0.193
100–129	1076	176	0.059	1.02 (0.85–1.22)	0.861	1.03 (0.86–1.23)	0.757	1.09 (0.91–1.31)	0.347
130–159	369	72	0.072	1.29 (1.00–1.66)	0.053	1.28 (1.00–1.66)	0.055	1.36 (1.05–1.75)	0.019
≥160	173	39	0.081	1.45 (1.04–2.02)	0.027	1.49 (1.07–2.08)	0.018	1.56 (1.12–2.18)	0.009
Cardiovascular death									
<70	2426	201	0.031	1 (Ref.)		1 (Ref.)		1 (Ref.)	
70–99	2234	176	0.029	0.93 (0.76–1.14)	0.493	0.93 (0.76–1.14)	0.502	0.98 (0.80–1.20)	0.819
100–129	1076	74	0.024	0.81 (0.62–1.06)	0.130	0.82 (0.62–1.07)	0.148	0.86 (0.65–1.12)	0.258
130–159	369	31	0.030	1.07 (0.73–1.57)	0.719	1.06 (0.72–1.56)	0.755	1.10 (0.75–1.62)	0.621
≥160	173	15	0.029	1.02 (0.61–1.73)	0.931	1.02 (0.60–1.73)	0.934	1.04 (0.61–1.77)	0.882
Myocardial infarction									
<70	2426	113	0.018	1 (Ref.)		1 (Ref.)		1 (Ref.)	
70–99	2234	129	0.022	1.31 (1.02–1.69)	0.038	1.33 (1.03–1.72)	0.027	1.43 (1.11–1.84)	0.006
100–129	107	60	0.020	1.30 (0.94–1.78)	0.110	1.33 (0.97–1.83)	0.082	1.44 (1.05–1.98)	0.025
130–159	369	24	0.023	1.63 (1.05–2.55)	0.031	1.65 (1.06–2.58)	0.027	1.81 (1.16–2.83)	0.010
≥160	173	15	0.030	2.03 (1.18–3.49)	0.010	2.13 (1.24–3.67)	0.006	2.25 (1.31–3.88)	0.004
Ischemic stroke									
<70	2426	142	0.022	1 (Ref.)		1 (Ref.)		1 (Ref.)	
70–99	2234	137	0.023	1.01 (0.80–1.28)	0.919	1.01 (0.80–1.28)	0.942	1.08 (0.85–1.37)	0.526
100–129	1076	69	0.023	1.04 (0.77–1.39)	0.803	1.05 (0.78–1.41)	0.739	1.11 (0.83–1.49)	0.485
130–159	369	28	0.027	1.29 (0.85–1.94)	0.227	1.27 (0.84–1.92)	0.255	1.34 (0.89–2.02)	0.166
≥160	173	15	0.030	1.43 (0.84–2.45)	0.187	1.46 (0.85–2.49)	0.168	1.54 (0.90–2.64)	0.114

CI, confidence interval; HR, hazard ratio; LDL-C, low-density lipoprotein cholesterol; PY, person-years; Ref, reference. Model 1: adjusted for age and sex. Model 2: adjusted for age, sex, body mass index, smoking status, alcohol consumption, exercise, and household income. Model 3: adjusted for age, sex, body mass index, smoking status, alcohol consumption, exercise, household income, Charlson Comorbidity Index, hypertension, diabetes mellitus, and antiplatelet agent use.

## Data Availability

This study used data from the NHIS of Korea. The data are not publicly available but can be accessed through the NHIS data request system for approved research purposes (https://nhiss.nhis.or.kr, accessed on 6 July 2025).

## Supplementary Materials

The following supporting information can be downloaded at https://www.mdpi.com/article/10.3390/jcm14144845/s1: Figure S1: Restricted cubic spline curves illustrating the continuous relationships between LDL-C levels and cardiovascular outcomes: (A) Cardiovascular death. (B) Myocardial infarction. (C) Ischemic stroke. LDL-C, low-density lipoprotein cholesterol. Table S1: Codes used to define study populations, comorbidities, medication history, and outcomes. Table S2: Clinical outcomes across LDL-C categories with an additional <55 mg/dL cut-off in the primary cohort (statin non-users). Table S3: Clinical outcomes across LDL-C categories with an additional <55 mg/dL cut-off in the secondary cohort (statin users).

## Abbreviations

The following abbreviations are used in this manuscript:

4D	Die Deutsche Diabetes Dialyse Studie
ASCVD	Atherosclerotic cardiovascular disease
AURORA	A Study to Evaluate the Use of Rosuvastatin in Subjects on Regular Hemodialysis: An Assessment of Survival and Cardiovascular Events
BMI	Body mass index
CAD	Coronary artery disease
CI	Confidence interval
CKD	Chronic kidney disease
DBP	Diastolic blood pressure
DM	Diabetes mellitus
eGFR	Estimated glomerular filtration rate
ESKD	End-stage kidney disease
HR	Hazard ratio
HTN	Hypertension
ICD-10	International Classification of Diseases, 10th Revision
LDL-C	Low-density lipoprotein cholesterol
MI	Myocardial infarction
NHIS	National Health Insurance Service
SBP	Systolic blood pressure
SHARP	Study of Heart and Renal Protection
TG	Triglyceride
