# Supplementary material for: Associations Between Low-Density Lipoprotein Cholesterol Levels and Cardiovascular Outcomes in Patients Undergoing Dialysis: A Nationwide Cohort Study
Source: J Clin Med. 2025 Jul 8;14(14):4845. doi: 10.3390/jcm14144845 (PMC12295821; doi:10.3390/jcm14144845)
Supplement: Supplementary file 1 [file jcm-14-04845-s001.zip › jcm-3702378-supplementary.pdf]

Supplementary Materials

Table S1. Codes used to define study populations, comorbidities, medication history, and outcomes.

Diagnosis	Definition
Haemodialysis	Defined as procedure codes O7020, O7021, or O9991
Peritoneal dialysis	Defined as procedure code O707
Myocardial infarction	Hospital admission with ICD-10 codes I21 or I22 as the primary diagnosis, accompanied by a coronary revascularization procedure code
Coronary artery bypass graft surgery	Defined as procedure codes O1640–O1642, O1647–O1649, OA640–OA642, or OA647–OA649
Percutaneous coronary intervention	Defined as procedure codes M6551–M6554, M6561–M6567, M6571, M6572, or O1876–O1877
Coronary angiography	Defined as procedure code HA670
Ischemic stroke	Hospital admission with ICD-10 codes I63 or I64 as the primary diagnosis, accompanied by a brain imaging procedure code
Brain imaging	Defined as procedure codes HA441, HA451, HA461, HA851, HE101, HE201, HE301, HE401, HE501, HE102, HE135, HE136, HE202, HE235, HE236, HE301, HE302, HE501, HE502, HE535, HE536, HI101, HI135, HI136, HI201, HI235, HI236, HI301, HI401, HI501, HI535, HI536, HJ101, HJ135, HJ136, HJ201, HJ235, HJ236, HJ301, HJ401, HJ501, HJ535, or HJ536
Coronary artery disease	Defined as the absence of diagnostic codes for myocardial infarction and the presence of a coronary revascularization procedure code
Prior kidney transplantation	Defined as procedure code R3280
Hypertension	Defined as ICD-10 codes I10–13 and I15 and prescription of antihypertensive agents
Diabetes mellitus	Defined as ICD-10 codes E10–14 and prescription of antidiabetic agents
**Cardiovascular death**	Defined as death due to cardiovascular disease (ICD-10 codes I00–I99)
**Medications**	

Statin	Rosuvastatin, atorvastatin, simvastatin, pitavastatin, pravastatin, fluvastatin, lovastatin
Other lipid-lowering drugs	evolocumab, alirocumab, ezetimibe, fenofibrate, bezafibrate, ciprofibrate, etofibrate, gemfibrozil, omega-3, nicotinic acid, acipimox
Antihypertensive agents	Amlodipine, nifedipine, nilvadipine, nimodipine, barnidipine, benidipine, cilnidipine, felodipine, isradipine, lacidipine, lercanidipine, manidipine, moxonidine, nicardipine, nitrendipine, nisoldipine, efonidipine, verapamil, diltiazem, azilsartan, candesartan, eprosartan, fimasartan, irbesartan, losartan, olmesartan, telmisartan, valsartan, alacepril, benazepril, captopril, cilazapril, enalapril, fosinopril, imidapril, lisinopril, moexipril, perindopril, ramipril, temocapril, zofenopril, carvedilol, atenolol, nebivolol, bisoprolol, metoprolol, amosulalol, arotinolol, betaxolol, bevantolol, celiprolol, labetalol, nadolol, propranolol, hydrochlorothiazide, chlorthalidone, tripamide, spironolactone, amiloride, furosemide, azosemide, torasemide, xipamide, metolazone, prazosin, cicletanine, clonidine, minoxidil, cadralazine
Antidiabetic agents	Metformin, gemigliptin, sitagliptin, vildagliptin, saxagliptin, linagliptin, teneligliptin, alogliptin, anagliptin, evogliptin, dapagliflozin, empagliflozin, ipragliflozin, ertugliflozin, enavogliflozin, acarbose, miglitol, voglibose, glibenclamide, gliclazide, glimepiride, glipizide, gliquidone, rosiglitazone, pioglitazone, lobeglitazone, repaglinide, nateglinide, mitiglinide, exenatide, lixisenatide, dulaglutide, albiglutide, insulin
Antiplatelet agent	Aspirin, clopidogrel, prasugrel, ticagrelor, cilostazol, dipyridamole, ticlopidine
Anticoagulants	Warfarin, rivaroxaban, dabigatran, apixaban, edoxaban

Abbreviations: ICD-10, International Classification of Diseases-10th revision.

Figure S1. Restricted cubic spline curves illustrating the continuous relationships between LDL-C levels and cardiovascular outcomes. (A) Cardiovascular death. (B) Myocardial infarction. (C) Ischemic stroke. LDL-C, low-density lipoprotein cholesterol.

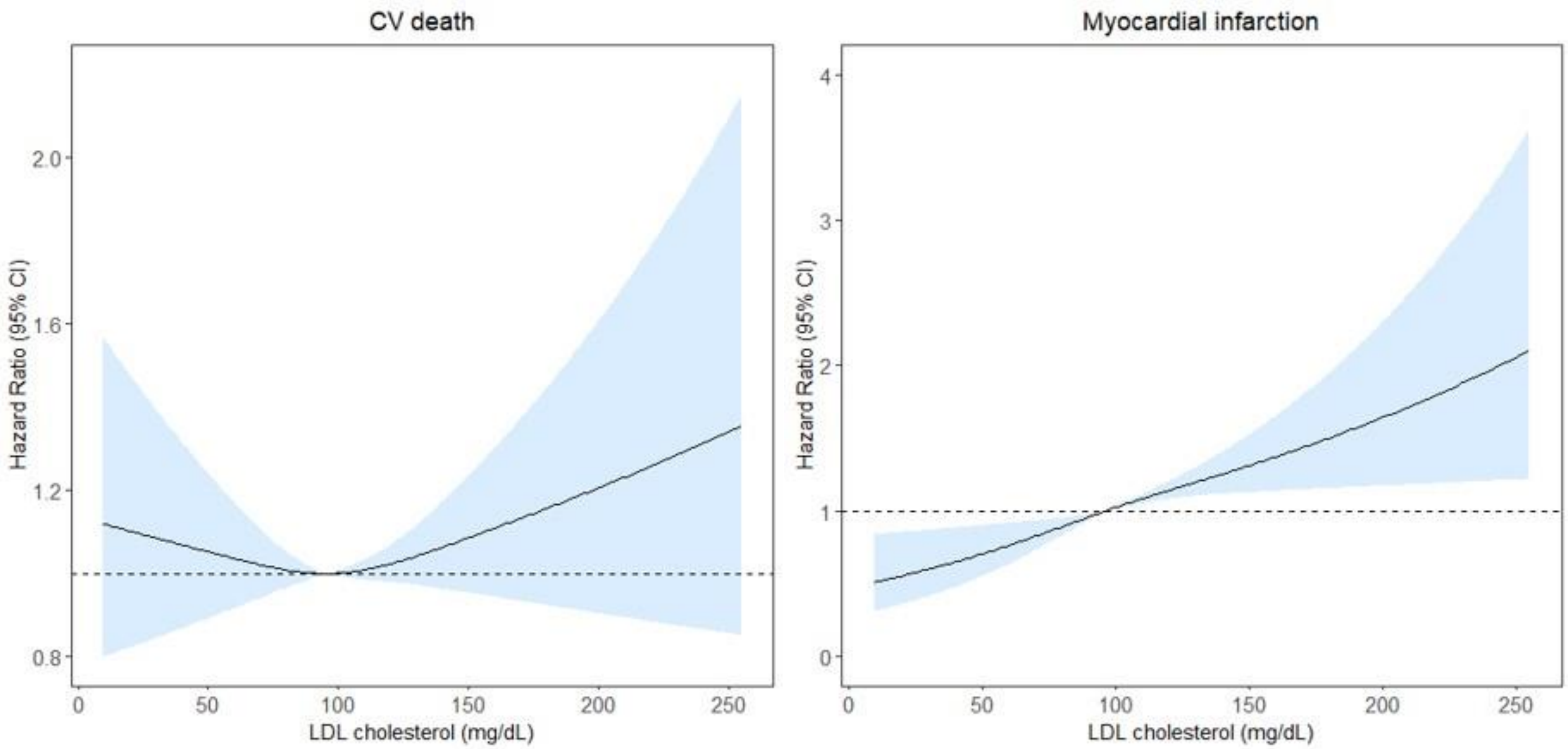

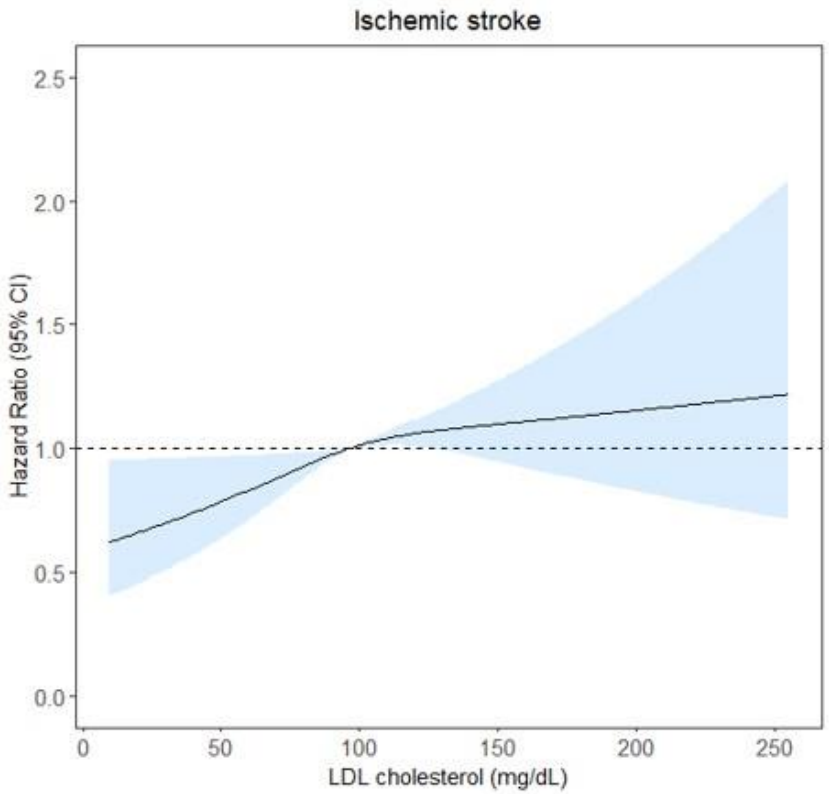

Ischemic stroke
Hazard Ratio (95% CI)
2.5
2.0
1.5
1.0
0.5
0.0
0
50
100
150
200
250
LDL cholesterol (mg/dL)

Table S2. Clinical outcomes across LDL-C categories with an additional <55 mg/dL cut-off in the primary cohort (statin non-users)

Total patients (N=15,414)				Model 1		Model 2		Model 3	
LDL-C categories	Participants	Events	Incidence rate (per 1,000 PY)	HR (95% CI)	p-value	HR (95% CI)	p-value	HR (95% CI)	p-value
**Composite outcome (Cardiovascular death, myocardial infarction, and ischemic stroke)**									
<55	1,051	129	0.046	1 (Ref.)		1 (Ref.)		1 (Ref.)	
55–69	1,866	254	0.049	1.02 (0.82–1.26)	0.863	1.05 (0.85–1.30)	0.669	1.06 (0.86–1.32)	0.564
70–99	5,459	823	0.054	1.08 (0.90–1.31)	0.397	1.12 (0.93–1.35)	0.220	1.16 (0.97–1.40)	0.112
100–129	4,541	755	0.059	1.17 (0.97–1.41)	0.099	1.21 (1.00–1.46)	0.048	1.28 (1.06–1.54)	0.011
130–159	1,797	316	0.063	1.27 (1.04–1.56)	0.022	1.33 (1.08–1.63)	0.008	1.37 (1.11–1.69)	0.003
≥160	700	125	0.066	1.37 (1.07–1.76)	0.012	1.41 (1.10–1.81)	0.006	1.49 (1.16–1.91)	0.002
**Cardiovascular death**									
<55	1,051	81	0.029	1 (Ref.)		1 (Ref.)		1 (Ref.)	
55–69	1,866	121	0.023	0.75 (0.56–0.99)	0.043	0.78 (0.59–1.03)	0.078	0.78 (0.59–1.04)	0.088
70–99	5,459	418	0.026	0.84 (0.66–1.07)	0.155	0.89 (0.70–1.13)	0.323	0.91 (0.71–1.15)	0.426
100–129	4,541	353	0.026	0.82 (0.65–1.05)	0.114	0.87 (0.68–1.11)	0.270	0.90 (0.71–1.15)	0.417
130–159	1,797	147	0.028	0.88 (0.67–1.16)	0.376	0.96 (0.73–1.26)	0.757	0.98 (0.74–1.29)	0.888
≥160	700	58	0.029	0.94 (0.67–1.32)	0.733	1.02 (0.72–1.43)	0.927	1.05 (0.75–1.48)	0.781
**Myocardial infarction**									
<55	1,051	28	0.010	1 (Ref.)		1 (Ref.)		1 (Ref.)	
55–69	1,866	64	0.012	1.19 (0.76–1.86)	0.439	1.24 (0.79–1.93)	0.353	1.24 (0.79–1.93)	0.347
70–99	5,459	202	0.013	1.26 (0.85–1.87)	0.252	1.30 (0.88–1.93)	0.193	1.34 (0.90–1.99)	0.152
100–129	4,541	223	0.017	1.67 (1.13–2.48)	0.011	1.69 (1.14–2.51)	0.009	1.78 (1.20–2.64)	0.004
130–159	1,797	87	0.017	1.73 (1.13–2.66)	0.012	1.71 (1.12–2.63)	0.014	1.75 (1.14–2.69)	0.011
≥160	700	49	0.025	2.65 (1.66–4.22)	<.001	2.55 (1.60–4.08)	<.001	2.60 (1.62–4.16)	<.001

**Ischemic stroke**									
<55	1,051	33	0.012	1 (Ref.)		1 (Ref.)		1 (Ref.)	
55–69	1,866	98	0.019	1.52 (1.02–2.25)	0.039	1.55 (1.04–2.30)	0.031	1.58 (1.07–2.35)	0.023
70–99	5,459	296	0.019	1.50 (1.05–2.15)	0.027	1.55 (1.08–2.22)	0.018	1.62 (1.13–2.33)	0.009
100–129	4,541	275	0.021	1.62 (1.13–2.33)	0.009	1.65 (1.15–2.38)	0.007	1.78 (1.24–2.56)	0.002
130–159	1,797	114	0.022	1.73 (1.17–2.55)	0.006	1.77 (1.20–2.62)	0.004	1.85 (1.25–2.73)	0.002
≥160	700	44	0.022	1.79 (1.14–2.82)	0.012	1.82 (1.15–2.86)	0.010	1.93 (1.23–3.05)	0.005

CI, confidence interval; HR, hazard ratio; LDL-C, low-density lipoprotein cholesterol; PY, person-years; Ref, reference.

Model 1: adjusted for age and sex.

Model 2: adjusted for age, sex, body mass index, smoking status, alcohol consumption, exercise, and household income.

Model 3: adjusted for age, sex, body mass index, smoking status, alcohol consumption, exercise, household income, Charlson Comorbidity Index, hypertension, diabetes mellitus, and antiplatelet agent use.

Table S3. Clinical outcomes across LDL-C categories with an additional <55 mg/dL cut-off in the secondary cohort (statin users)

**Total patients (N=6,278)**				**Model 1**		**Model 2**		**Model 3**	
**LDL-C categories**	**Participants**	**Events**	**Incidence rate (per 1,000 PY)**	**HR (95% CI)**	**p-value**	**HR (95% CI)**	**p-value**	**HR (95% CI)**	**p-value**
**Composite outcome (Cardiovascular death, myocardial infarction, and ischemic stroke)**									
<55	1,164	181	0.062	1 (Ref.)		1 (Ref.)		1 (Ref.)	
55–69	1,262	213	0.065	1.05 (0.86–1.28)	0.613	1.07 (0.88–1.30)	0.523	1.11 (0.91–1.36)	0.292
70–99	2,234	375	0.064	1.06 (0.89–1.27)	0.513	1.07 (0.90–1.29)	0.437	1.16 (0.97–1.39)	0.097
100–129	1,076	176	0.059	1.05 (0.85–1.29)	0.683	1.07 (0.86–1.32)	0.555	1.16 (0.94–1.43)	0.181
130–159	369	72	0.072	1.32 (1.00–1.74)	0.049	1.33 (1.01–1.75)	0.044	1.44 (1.09–1.90)	0.011
≥160	173	39	0.081	1.49 (1.05–2.11)	0.024	1.55 (1.09–2.19)	0.015	1.65 (1.17–2.35)	0.005
**Cardiovascular death**									
<55	1,164	94	0.031	1 (Ref.)		1 (Ref.)		1 (Ref.)	
55–69	1,262	107	0.031	1.01 (0.76–1.33)	0.955	1.02 (0.77–1.34)	0.908	1.06 (0.80–1.40)	0.676
70–99	2,234	176	0.029	0.94 (0.73–1.20)	0.603	0.94 (0.73–1.21)	0.636	1.01 (0.78–1.30)	0.953
100–129	1,076	74	0.024	0.82 (0.60–1.11)	0.195	0.83 (0.61–1.13)	0.227	0.88 (0.65–1.21)	0.432
130–159	369	31	0.030	1.08 (0.71–1.63)	0.722	1.07 (0.71–1.62)	0.740	1.14 (0.75–1.72)	0.541
≥160	173	15	0.029	1.03 (0.60–1.78)	0.921	1.03 (0.60–1.79)	0.912	1.07 (0.62–1.86)	0.799
**Myocardial infarction**									
<55	1,164	52	0.017	1 (Ref.)		1 (Ref.)		1 (Ref.)	
55–69	1,262	61	0.018	1.09 (0.75–1.58)	0.653	1.11 (0.76–1.60)	0.593	1.17 (0.81–1.69)	0.415
70-99	2,234	129	0.022	1.37 (0.99–1.89)	0.057	1.41 (1.02–1.95)	0.040	1.55 (1.12–2.14)	0.008
100–129	1,076	60	0.020	1.36 (0.93–1.98)	0.112	1.40 (0.96–2.04)	0.080	1.56 (1.07–2.28)	0.021
130–159	369	24	0.023	1.71 (1.05–2.79)	0.032	1.75 (1.07–2.85)	0.026	1.96 (1.20–3.21)	0.007
≥160	173	15	0.030	2.13 (1.19–3.79)	0.011	2.25 (1.26–4.02)	0.006	2.44 (1.37–4.37)	0.003

**Ischemic stroke**									
<55	1,164	67	0.023	1 (Ref.)		1 (Ref.)		1 (Ref.)	
55–69	1,262	75	0.022	0.98 (0.70–1.36)	0.899	0.98 (0.70–1.36)	0.890	1.02 (0.73–1.41)	0.928
70–99	2,234	137	0.023	1.00 (0.75–1.34)	0.996	1.00 (0.74–1.34)	0.980	1.09 (0.81–1.46)	0.574
100–129	1,076	69	0.023	1.03 (0.73–1.45)	0.882	1.04 (0.74–1.46)	0.831	1.12 (0.79–1.58)	0.520
130–159	369	28	0.027	1.27 (0.81–1.99)	0.290	1.25 (0.80–1.96)	0.323	1.35 (0.86–2.11)	0.192
≥160	173	15	0.030	1.42 (0.81–2.49)	0.226	1.44 (0.82–2.53)	0.206	1.56 (0.88–2.74)	0.126

CI, confidence interval; HR, hazard ratio; LDL-C, low-density lipoprotein cholesterol; PY, person-years; Ref, reference.

Model 1: adjusted for age and sex.

Model 2: adjusted for age, sex, body mass index, smoking status, alcohol consumption, exercise, and household income.

Model 3: adjusted for age, sex, body mass index, smoking status, alcohol consumption, exercise, household income, Charlson Comorbidity Index, hypertension, diabetes mellitus, and antiplatelet agent use.
